# Bridgehead vicinal diallylation of norbornene derivatives and extension to propellane derivatives via ring-closing metathesis

**DOI:** 10.3762/bjoc.12.177

**Published:** 2016-08-22

**Authors:** Sambasivarao Kotha, Rama Gunta

**Affiliations:** 1 Department of Chemistry, Indian Institute of Technology-Bombay, Powai, Mumbai, India

**Keywords:** norbornene, propellane derivatives, ring-closing metathesis, single-crystal X-ray diffraction, vicinal diallylation

## Abstract

Here, we report a simple synthetic strategy to the bridgehead vicinal diallylation of norbornene derivatives. These substrates are useful to generate propellanes via ring-closing metathesis. Single-crystal X-ray diffraction analysis of four compounds led to the realization of configurational correction of earlier reported molecules.

## Introduction

The norbornene moiety is a useful template and also a versatile synthon in organic synthesis [1]. The double bond present in the norbornene frame is strained and therefore participates in cycloaddition sequences as a C_2_-synthon [2–3]. It was reported that the norbornene system is as strained as cyclopropane or cyclobutane (norbornene, 100 kJ/mol; cyclopropane, 115 kJ/mol; cyclobutane, 110 kJ/mol) [4–5]. Some of the annulated norbornene derivatives undergo retro Diels–Alder (rDA) reactions at ambient temperature in the presence of methylaluminium dichloride and a reactive dienophile [6–8]. Cage compounds with interesting applications have been assembled by a cyclization reaction starting with suitably functionalized norbornene derivatives [9–11]. Moreover, the norbornene unit induces a hairpin-like architecture when it is incorporated into a peptide chain. This property is useful to design norbornene-based ionophores [12]. Due to the strained nature of norbornene systems they are useful precursors for ring-rearrangement metathesis (RRM) [13–21] to generate intricate polycyclics involving non-traditional retrosynthetic routes. Recently, functionalization of unactivated aromatic C–H bonds was achieved by using palladium catalysts and norbornene (Catellani reaction) [22–23]. In view of these applications, the design and synthesis of vicinal diallylnorbornene derivatives is a worthwhile exercise. The double bond present in the allyl group can be further converted into various other useful functionalities for further synthetic manipulation by adopting the appropriate functional group transformations.

### Strategy

**Figure 1:**
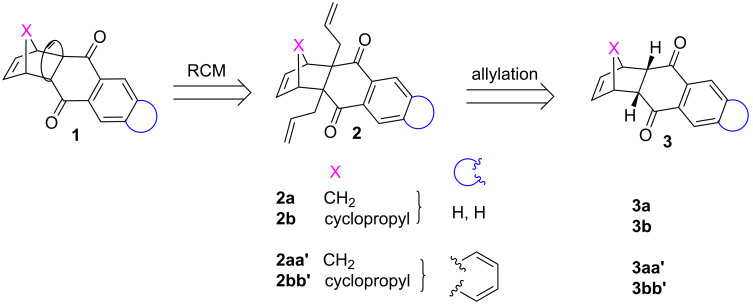
Retrosynthetic approach to propellane derivatives.

Our approach to various propellane derivatives is shown in Figure 1. The target propellane **1** could be assembled from diallyl compound **2** via ring-closing metathesis (RCM) [24–32]. Whereas, the diallyl derivative **2** can be derived from a readily available Diels–Alder (DA) adduct **3** through an allylation sequence.

## Results and Discussion

**Scheme 1:**
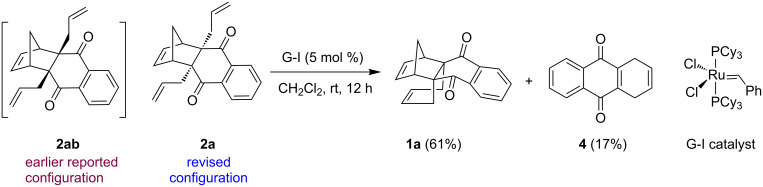
Synthesis of the propellane derivative **1a** via RCM.

Installation of two C–C bonds to generate quaternary centers in a stereocontrolled manner in a single step is not a trivial exercise. Generally, it was accomplished by radical three-component coupling reactions or Michael-type additions of organocopper reagents starting with conjugated carbonyl compounds [33–34]. But, the resulting alkyl groups are in *trans* orientation. Our journey to propellane **1** synthesis (Figure 1) was commenced with the preparation of known DA adducts **3a**, **3b**, **3aa'** and **3bb'** [35–37]. In this regard, DA adduct **3a** was treated with allyl bromide in the presence of NaH to obtain the corresponding *O*-allylated compound (70%) and *C*-allylated compound **2a** (28%) by using our earlier reported method [38]. Next, diallyl compound **2a** on RCM using Grubbs first generation (G-I) catalyst in CH_2_Cl_2_ at room temperature (rt) gave the desired propellane derivative **1a** (61%) along with a minor amount of quinone derivative **4** (17%) (Scheme 1). The formation of quinone **4** can be explained on the basis that compound **2a** underwent rDA and RCM in one-pot. Here, the compound **2a** didn’t undergo RRM because a metallacyclobutane cannot be formed between the allyl and norbornene double bonds due to structural constraint [39] and moreover, we didn’t observe any ring-opening metathesis (ROM) product during RCM reaction. This may be due to the fact that sparging with an inert gas (N_2_ or Ar) during RCM process helps to accelerate the loss of ethylene and thus, prevents ROM [39].

**Scheme 2:**
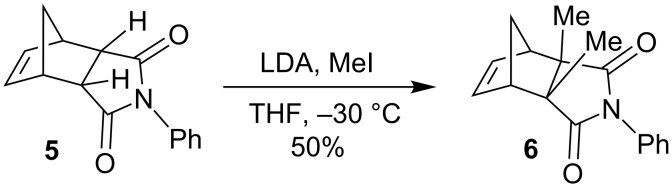
Garratts work on alkylation of norbornene with retention of configuration.

Garratt and Hollowood reported that bridgehead functionalization of norbornene derivatives such as *endo*-5-norbornene-2,3-dicarboximide **5** gave bridgehead alkylated compound **6** with retention of configuration (Scheme 2) [40]. Based on this report, we expected the allyl groups introduced via alkylation sequence will occupy the *exo* position (see **2ab**) because the bridgehead hydrogens in DA adduct **3a** are in *exo* configuration. Thus, in the final compound **1a** the newly formed 6-membered ring during RCM is supposed to be in the *exo* configuration.

**Figure 2:**
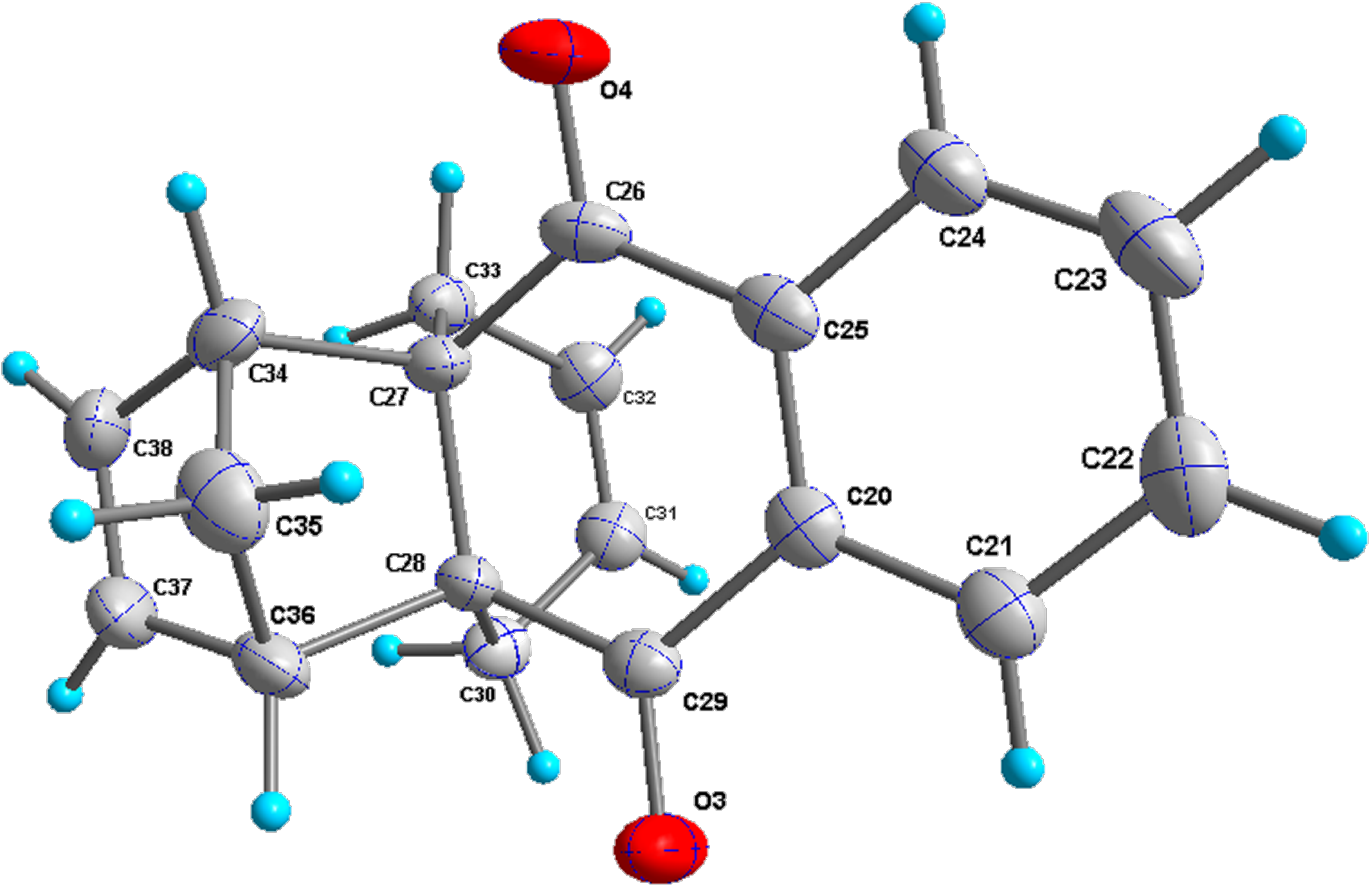
The molecular structure of **1a**, with displacement ellipsoids drawn at the 50% probability level.

To our surprise, single-crystal X-ray analysis of **1a** revealed that the 6-membered ring (C28–C30–C31–C32–C33–C27) formed via RCM is in *endo* configuration as depicted in Figure 2.

**Scheme 3:**
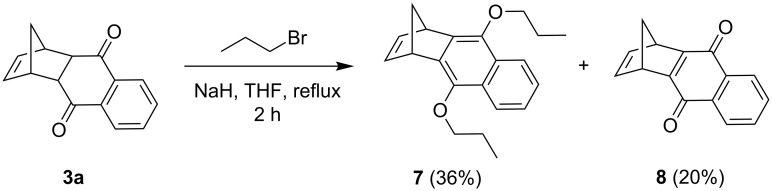
Control experiment carried out to probe the configuration of **2a**.

At this point, we turned our attention to understand the configurational origin of the allyl groups in **2a**. To understand whether compound **2a** was formed by Claisen rearrangement (CR) of the corresponding *O*-allyl compound or by carbanion mediated *C*-allylation of the DA adduct **3a**, we carried out the alkylation of compound **3a** with *n*-propyl bromide in refluxing THF for 2 h. Here, di-*O*-propyl compound **7** was obtained in 36% yield along with a dehydrogenated compound **8** (20%, Scheme 3). Surprisingly, no *C*-alkylation product was observed from **3a**. This result suggests that the *C*-allyl compound **2a** was formed from the corresponding *O*-allyl compound via CR.

**Figure 3:**
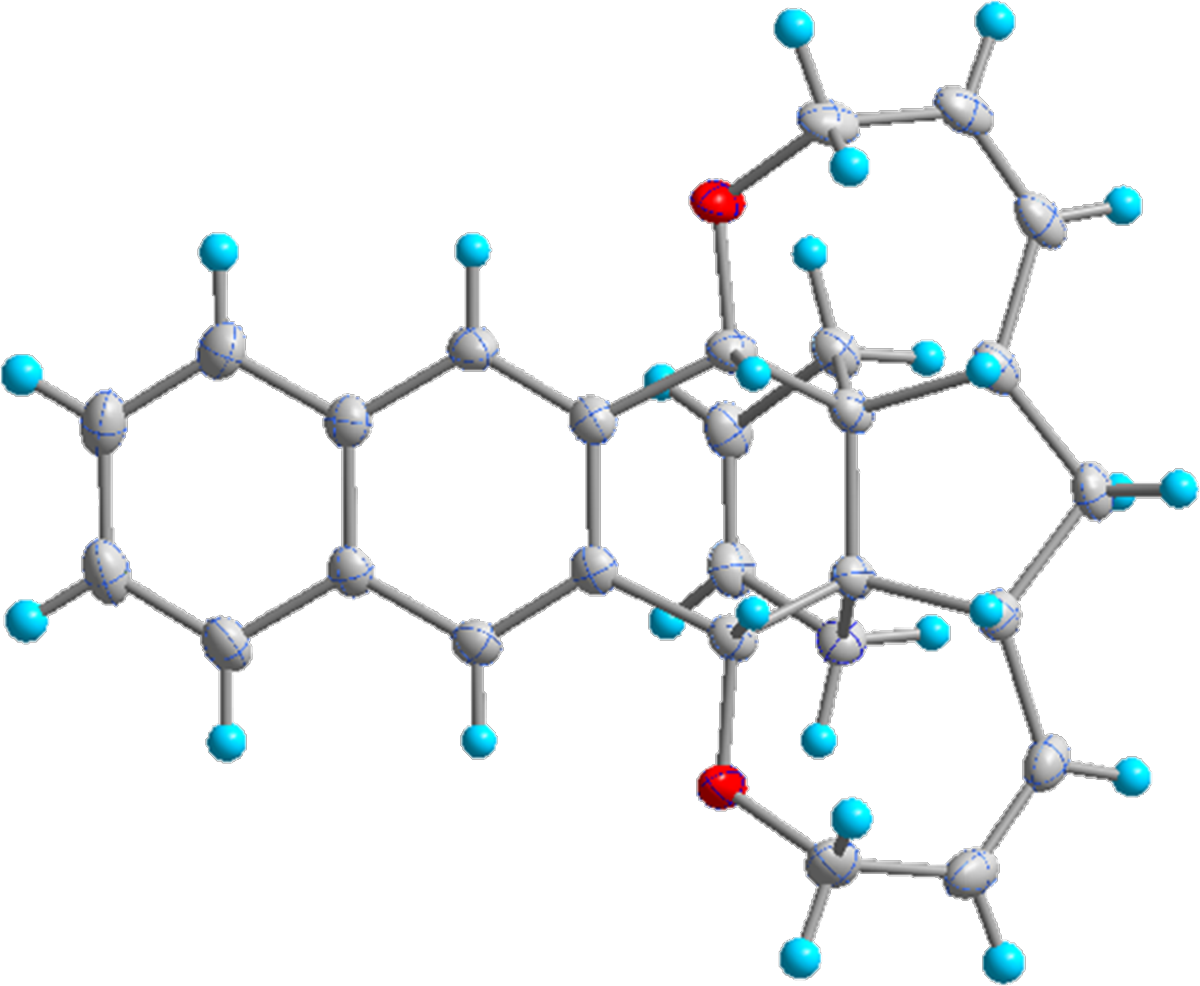
Crystal structure of compound **15** showing 50% displacement ellipsoids.

Based on the X-ray structure of **1a** and the above observations, it is clear that the allyl groups in **2a** are in *endo* configuration which can be explained as follows. Since the stereocenters are unaffected during the RCM sequence it is evident that the allyl groups present in **2a** should be in *endo* configuration. To confirm the configuration of the allyl groups, the X-ray structure of previously reported oxa-bowl/propellane hybrid (**15**) [38] was also recorded and it is in agreement with the above findings (Figure 3). These results suggested the revision of earlier reported configuration of allyl groups. More specifically, various compounds (**2ab**, **2aa'b** and **9a–15a**) reported in our previous report [38] need configurational correction and the revised structures (**2aa'** and **9–15**) are included in Table 1.

**Table 1:** Revised structures from our previous work [38] with correct configuration.

Entry	Revised structures	Earlier reported structures (ref. [38])	Entry	Revised structures	Earlier reported structures (ref. [38])

1	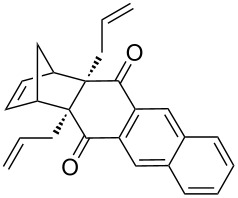 **2aa’**	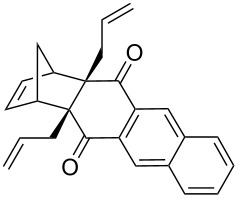 **2aa’b**	5	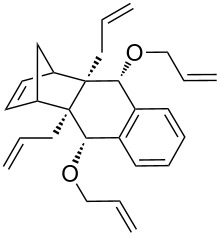 **12**	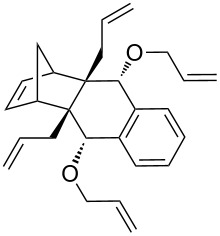 **12a**
2	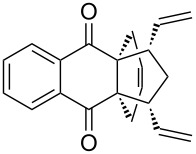 **9**	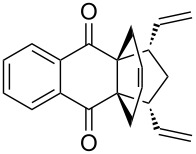 **9a**	6	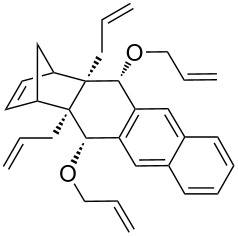 **13**	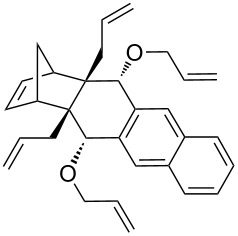 **13a**
3	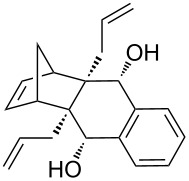 **10**	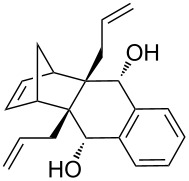 **10a**	7	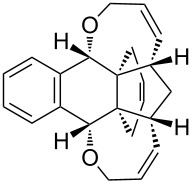 **14**	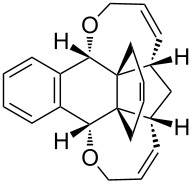 **14a**
4	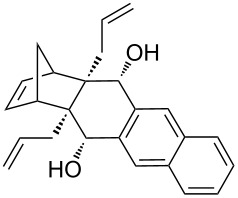 **11**	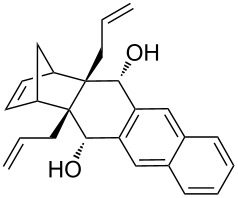 **11a**	8	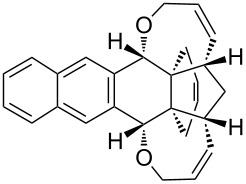 **15**	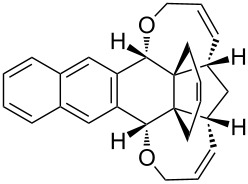 **15a**

**Scheme 4:**
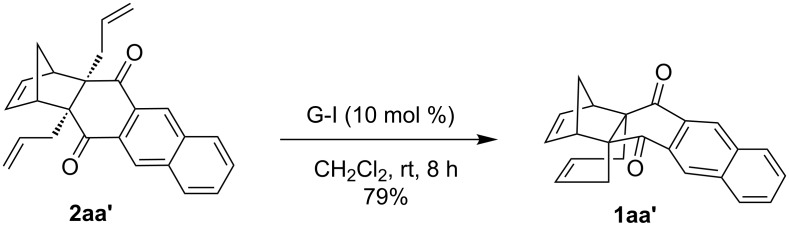
RCM of the compound **2aa'**.

When the other previously prepared diallyl compound **2aa'** [38] was subjected to RCM using G-I catalyst under similar reaction conditions the propellane derivative **1aa'** was obtained in 79% yield (Scheme 4).

**Scheme 5:**
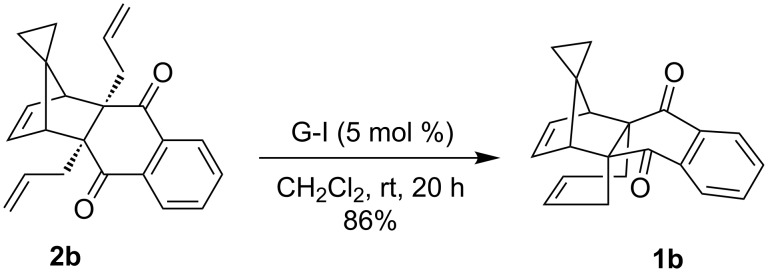
RCM approach to the propellane derivative **1b**.

To expand the scope of this strategy, cyclopropyl containing diallyl products **2b** and **2bb'** were also prepared along similar lines starting with the corresponding DA adducts **3b** and **3bb'** [41]. Initially, the diallyl compound **2b** was reacted with G-I catalyst to afford the desired propellane **1b** in 86% yield (Scheme 5). Its structure has been established on the basis of spectroscopic data (^1^H NMR, ^13^C NMR and DEPT-135) and was further supported by HRMS data.

**Figure 4:**
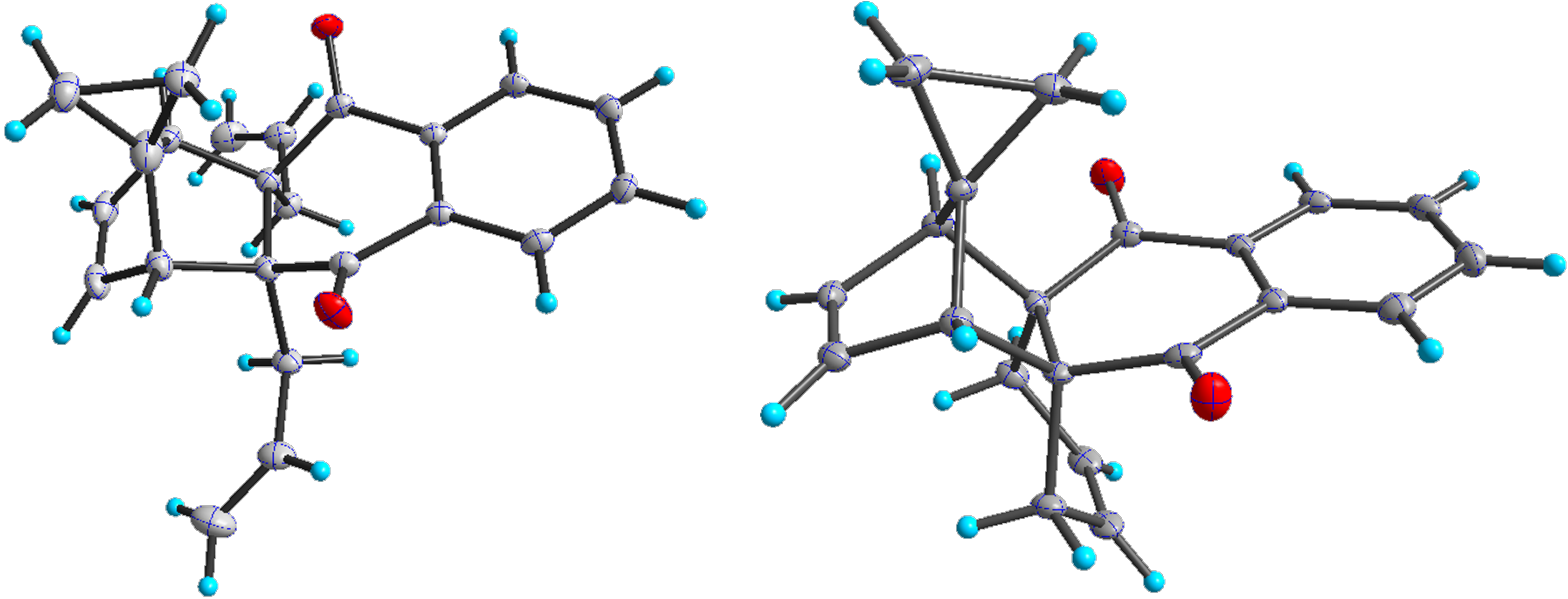
The molecular structures of the compounds **2b** (left) and **1b** (right) showing 30% displacement ellipsoids.

In addition, the configuration of **1b** and **2b** were unambiguously determined via single-crystal X-ray diffraction analysis (Figure 4). Based on this data it is clear that the bridgehead allyl groups in the RCM precursor **2b** are in *endo* configuration. Subsequent RCM of diallyl compound **2b** gave the ring-closing product **1b** with retention of the configuration.

**Scheme 6:**
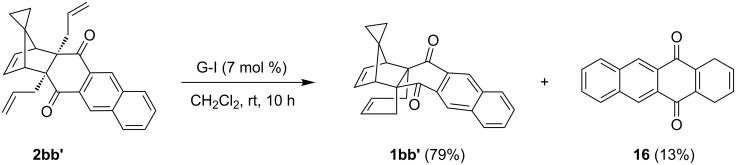
Construction of the propellane derivative **1bb'** using RCM.

Similarly, staring with substrate **2bb'**, another propellane derivative **1bb'** was synthesized using the same catalyst (i.e., G-I) in CH_2_Cl_2_ at rt. Here, along with the desired propellane **1bb'** (79%) a minor amount of quinone derivative **16** (13%) was also generated due to a one-pot RCM-rDA sequence of **2bb'** which is similar to the substrate **2a** (Scheme 6). Compound **1bb'** was characterized based on the ^1^H and ^13^C NMR, DEPT-135 and further supported by HRMS data. However, spectroscopic data of quinone **16** were identical with the literature values [41].

## Conclusion

This methodology was found to be useful to synthesize various propellane derivatives containing a norbornene moiety by employing RCM sequence. Moreover, we have firmly established the configuration of allyl groups at bridgehead position of norbornene derivatives by single-crystal X-ray diffraction analysis. A control experiment with propyl bromide provided an insight into the reaction mechanism that the bridgehead allylation proceeds through enolization, *O*-allylation followed by CR and not via carbanion chemistry. This alternative strategy is useful to introduce vicinal diallyl groups in a *cis* orientation to generate propellane derivatives, which is a different protocol from previously reported methods where the two vicinal alkyl groups are introduced in *trans* orientation. In this study, we have also revised the configuration of our earlier reported molecules containing allyl groups and oxa-bowl/propellane hybrids. Since non-flattened molecules are implicated in biological systems, our results would be useful in drug design [42].

## Supporting Information

Crystallographic data have been deposited with the Cambridge Crystallographic Data Centre (CCDC) as supplementary publication numbers CCDC 1475412 (**1a**), 1475453 (**1b**), 1475403 (**2b**) and 1451438 (**15**). Copies of the data can be obtained free of charge on application to the Director at CCDC, 12 Union Road, Cambridge CB2 1EZ, UK (FAX: (+44) 1223-336-033; email: deposit@ccdc.cam.ac.uk).

File 1 Experimental procedures, characterization data, copies of ^1^H & ^13^C NMR for all new compounds and X-ray data of the compounds **1a**, **1b**, **2b** and **15**.

File 2 Crystallographic information files of compounds **1a** (CCDC 1475412), **1b** (CCDC 1475453), **2b** (CCDC 1475403) and **15** (CCDC 1451438).
