# Supplementary material for: Bridgehead vicinal diallylation of norbornene derivatives and extension to propellane derivatives via ring-closing metathesis
Source: Beilstein J Org Chem. 2016 Aug 22;12:1877–83. doi: 10.3762/bjoc.12.177 (PMC5082723; doi:10.3762/bjoc.12.177)
Supplement: File 1 — Experimental procedures, characterization data, copies of 1H & 13C NMR for all new compounds and X-ray data of the compounds 1a, 1b, 2b and 15. [file Beilstein_J_Org_Chem-12-1877-s001.pdf]

Supporting Information

for

Bridgehead vicinal diallylation of norbornene derivatives and extension to propellane derivatives via ring-closing metathesis

Sambasivarao Kotha*[§] and Rama Gunta

Address: Department of Chemistry, Indian Institute of Technology-Bombay, Powai, Mumbai, India

[§]Fax: 022-25767152

Email: Sambasivarao Kotha - srk@chem.iitb.ac.in

*Corresponding author

Experimental procedures, characterization data, copies of ^{1}H & ^{13}C NMR for all new compounds and X-ray data of the compounds 1a, 1b, 2b and 15

Contents

1. Experimental procedures and characterization data S2–S5
2. Copies of ^{1}H and ^{13}C NMR spectra of the all new compounds S6–S14
3. X-ray data for the compounds **1a**, **1b**, **2b** and **15** S15–S18

1. Experimental procedures and characterization data

General methods and techniques used were described in our previous paper [1].

1.1. General procedure for ring-closing metathesis of *C*-allyl derivatives 2a, 2aa', 2b and 2bb': A solution of **2a**/**2aa'**/**2b**/**2bb'** in dry CH_2Cl_2 was degassed with nitrogen for 5 min. Next, Grubbs 1st generation (G-I) catalyst (5–10 mol %) was added and the resulting reaction mixture was stirred at room temperature (rt) for 8–20 h. After completion of the reaction (TLC/crude ^{1}H NMR), the solvent was removed and the crude product was purified by silica gel column chromatography using an appropriate mixture of EtOAc and petroleum ether as an eluent to obtain the desired propellane derivatives **1a**/**1aa'**/**1b**/**1bb'**.

Compound 1a: Yellow solid (333 mg, 61%), obtained from **2a** (600 mg, 1.971 mmol), G-I (81 mg, 5 mol %), CH_2Cl_2 (80 mL). The crude product was purified by silica gel column chromatography (2% EtOAc–petroleum ether) followed by recrystallization from a mixture of CH_2Cl_2 and petroleum ether. Mp: >160 ^oC (decomposed & colour changed to brown).

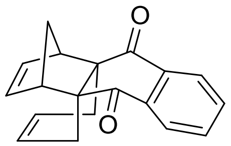

^{1}H NMR (400 MHz, $CDCl_3$): δ (ppm) = 8.06 (dd, J = 5.8, 3.4 Hz, 2H), 7.68 (dd, J = 5.8, 3.3 Hz, 2H), 6.43 (t, J = 2.0 Hz, 2H), 5.63 (t, J = 3.6 Hz, 2H), 3.38–3.36 (m, 2H), 2.79–2.77 (m, 1H), 2.75–2.73 (m, 1H), 2.08 (dd, J = 13.7, 0.4 Hz, 2H), 1.51 (dt, J = 9.2, 1.7 Hz, 1H), 1.34 (d, J = 9.2 Hz, 1H); ^{13}C NMR (100.6 MHz, $CDCl_3$): δ (ppm) = 202.5, 138.9, 136.6, 134.0, 129.2, 127.3, 62.3, 52.8, 47.8, 35.7; HRMS (ESI, Q-ToF) *m/z*: calculated for $C_{19}H_{16}NaO_2$ $[M+Na]^+$: 299.1043, found: 299.1041; IR (neat): υ_{max} = 3054, 2986, 2857, 1667, 1591, 1307, 1267, 1065, 949, 693 cm^{-1}.

Compound 1aa': Yellow solid (20.40 mg, 79%), obtained from **2aa'** (28 mg, 0.079 mmol), G-I (6.50 mg, 10 mol %), CH_2Cl_2 (20 mL). Product has same R_f value as that of the starting material.

Mp: >170 °C (decomposed & colour changed to brown). ^{1}H NMR (400 MHz, $CDCl_3$): δ (ppm) = 8.61 (s, 2H), 8.03 (dd, J = 6.2, 3.3 Hz, 2H), 7.65 (dd, J = 6.3, 3.2 Hz, 2H), 6.46 (t, J = 1.9 Hz, 2H), 5.61 (t, J = 3.6 Hz, 2H), 3.45–3.44 (m, 2H), 2.85–2.83 (m, 1H), 2.82–2.80 (m, 1H), 2.10 (d, J = 13.9 Hz, 2H), 1.53 (dt, J = 9.2, 1.7 Hz, 1H), 1.39 (d, J = 9.2 Hz, 1H); ^{13}C NMR (100.6 MHz, $CDCl_3$): δ (ppm) = 202.7 (s), 139.1 (d), 135.3 (s), 132.4 (s), 130.1 (d), 129.4 (d), 129.2 (d), 129.1 (d), 62.7 (s), 53.0 (d), 47.9 (t), 35.9 (t); HRMS (ESI, Q-ToF) *m/z*: calculated for $C_{23}H_{18}NaO_2$ $[M+Na]^+$: 349.1199, found: 349.1200; IR (neat): υ_{max} = 3046, 2961, 2865, 1673, 1620, 1456, 1266, 907 cm^{-1}.

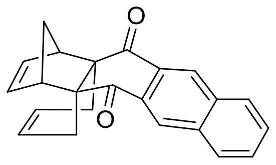

Compound 1b: White crystalline solid (19 mg, 86%), obtained from **2b** (24 mg, 0.073 mmol), G-I (3 mg, 5 mol %), CH_2Cl_2 (15 mL). Product has same R_f value as that of the starting material and the crude product was purified by silica gel column chromatography (2–3% EtOAc–petroleum ether) followed by recrystallization from a mixture of CH_2Cl_2 and petroleum ether. Mp: >197 °C (decomposed & slowly colour changed to brown).

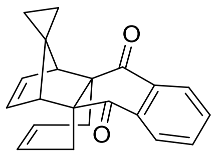

^{1}H NMR (400 MHz, $CDCl_3$): δ (ppm) = 8.00 (dd, J = 5.7, 3.3 Hz, 2H), 7.67 (dd, J = 5.8, 3.3 Hz, 2H), 6.51 (t, J = 1.9 Hz, 2H), 5.58 (t, J = 3.6 Hz, 2H), 3.00 (t, J = 1.8 Hz, 2H), 2.63–2.61 (m, 1H), 2.60–2.58 (m, 1H), 2.21 (d, J = 13.7 Hz, 2H), 0.43–0.40 (m, 2H), 0.08–0.04 (m, 2H); ^{13}C NMR (125.7 MHz, $CDCl_3$): δ (ppm) = 203.0 (s), 138.6 (d), 136.8 (s), 133.8 (d), 128.7 (d), 126.9 (d), 63.2 (s), 57.2 (d), 45.2 (s), 36.0 (t), 10.0 (t), 6.4 (t); HRMS (ESI, Q-ToF) *m/z*: calculated for $C_{21}H_{18}NaO_2$ $[M+Na]^+$: 325.1199, found: 325.1198; IR (neat): υ_{max} = 2979, 2867, 1669, 1592, 1300, 1276, 1034, 959, 695 cm^{-1}.

Compound 1bb': Light yellow solid (25 mg, 79%), obtained from **2bb'** (34 mg, 0.089 mmol), G-I (5.13 mg, 7 mol %), CH_2Cl_2 (35 mL). Product has same R_f value as that of the starting material and the crude product was purified by silica gel column chromatography (0.5% EtOAc–petroleum ether) followed by recrystallization from a mixture of CH_2Cl_2 and petroleum ether. Mp: >179 ^oC (decomposed & slowly colour changed to black).

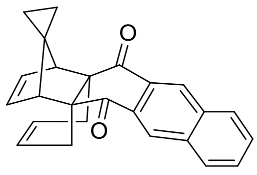

^1H NMR (400 MHz, $CDCl_3$): δ (ppm) = 8.56 (s, 2H), 8.03 (dd, J = 6.2, 3.3 Hz, 2H), 7.65 (dd, J = 6.3, 3.2 Hz, 2H), 6.54 (t, J = 2.0 Hz, 2H), 5.56 (t, J = 3.6 Hz, 2H), 3.08 (t, J = 2.0 Hz, 2H), 2.70–2.68 (m, 1H), 2.66–2.65 (m, 1H), 2.23 (d, J = 13.9 Hz, 2H), 0.47–0.43 (m, 2H), 0.10–0.07 (m, 2H); ^{13}C NMR (100.6 MHz, $CDCl_3$): δ (ppm) = 203.1 (s), 138.7 (d), 135.2 (s), 132.6 (s), 130. 0 (d), 129.3 (d), 128.7 (d), 128.6 (d), 63.6 (s), 57.5 (d), 45.5 (s), 36.2 (t), 10.0 (t), 6.8 (t); HRMS (ESI, Q-ToF) *m/z*: calculated for $C_{25}H_{20}NaO_2$ $[M+Na]^+$: 375.1356, found: 375.1353.

1.2. Alkylation of the DA adduct 3a with *n*-propyl bromide: A solution of the DA adduct **3a** (150 mg, 0.669 mmol) in THF (5 mL) was added to a suspension of NaH (10 equiv) in THF (5 mL) at rt. Next, *n*-propyl bromide solution (3 equiv, ~0.2 mL) was added to the reaction mixture and refluxed (70 ^oC) for 2 h. After completion of the reation (TLC), excess NaH was quenched by the addition of saturated NH_4Cl solution (5 mL). Then, the reaction mixture was extracted with EtOAc (3 × 20 mL), washed with brine solution, dried over anhydrous Na_2SO_4 and concentrated under reduced pressure. Finally, the crude product was purified by silica-gel column chromatography (1% EtOAc–petroleum ether) to get the *O*-propyl compound **7** (75 mg, 36%) as a pink liquid. Further elution of the column with 2–3% EtOAc–petroleum ether gave the quinone **8** (30 mg, 20%) as a yellow solid.

Compound 7: ^{1}H NMR (500 MHz, $CDCl_3$): δ (ppm) = 8.05 (dd, J = 6.3, 3.3 Hz, 2H), 7.43 (dd, J = 6.3, 3.2 Hz, 2H), 6.77 (t, J = 1.7 Hz, 2H), 4.29 (t, J = 1.6 Hz, 2H), 4.11–4.06 (m, 2H), 4.02–3.97 (m, 2H), 2.27 (d, J = 7.4 Hz, 1H), 2.18 (d, J = 7.5 Hz, 1H), 1.94–1.87 (m, 4H), 1.14 (t, J = 7.40 Hz, 6H); ^{13}C NMR (125.7 MHz, $CDCl_3$): δ (ppm) = 144.4 (s), 141.8 (d), 136.2 (s), 128.3 (s), 125.3 (d), 122.2 (d), 76.5 (t), 65.3 (t), 47.1 (d), 23.9 (t), 10.9 (q); HRMS (ESI, Q-ToF) m/z: calculated for $C_{21}H_{24}NaO_2$ $[M+Na]^+$: 331.1669, found: 331.1665; IR (neat): υ_{max} = 2961, 2877, 1455, 1337, 1084, 963 cm^{-1}.

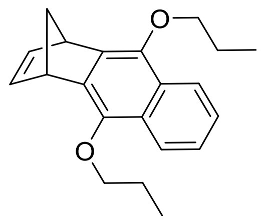

Compound 8: Mp: 140–142 ^{o}C; ^{1}H NMR (400 MHz, $CDCl_3$): δ (ppm) = 8.05–8.03 (m, 2H), 7.67–7.66 (m, 2H), 6.89 (t, J = 1.9 Hz, 2H), 4.24 (t, J = 1.6 Hz, 2H), 2.36, 2.31 (ABq, J_{AB} = 7.1 Hz, 2H); ^{13}C NMR (125.7 MHz, $CDCl_3$): δ (ppm) = 181.9 (s), 163.2 (s), 142.7 (d), 133.5 (d), 132.9 (s), 126.3 (d), 73.5 (t), 48.9 (d); HRMS (ESI, Q-ToF) m/z: calculated for $C_{15}H_{11}O_2$ $[M+H]^+$: 223,0754, found: 223,0755.

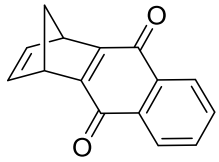

1.3. References

1. Kotha, S.; Gunta, R. *Beilstein J. Org. Chem.* **2015**, *11*, 1727–1731. doi: 10.3762/bjoc.11.188

2. Copies of ^{1}H and ^{13}C NMR spectra of the all new compounds

Compound **1a** (^{1}H NMR)

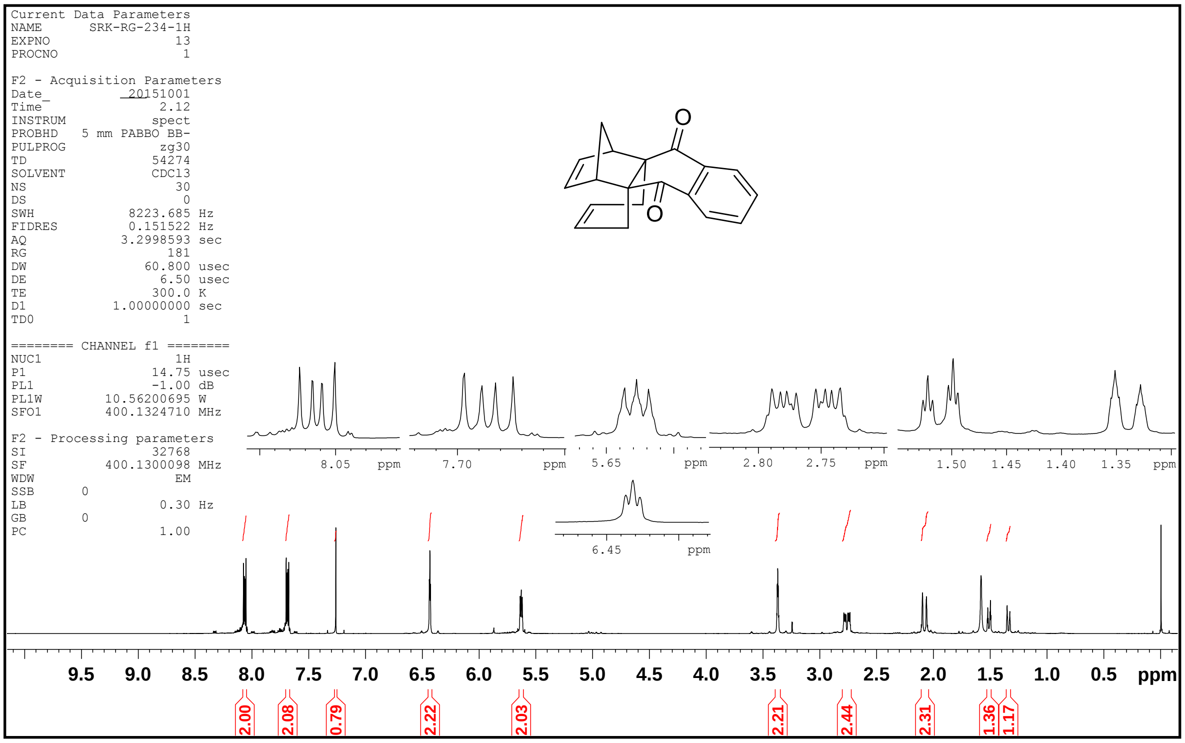

Compound **1a** (^{13}C NMR)

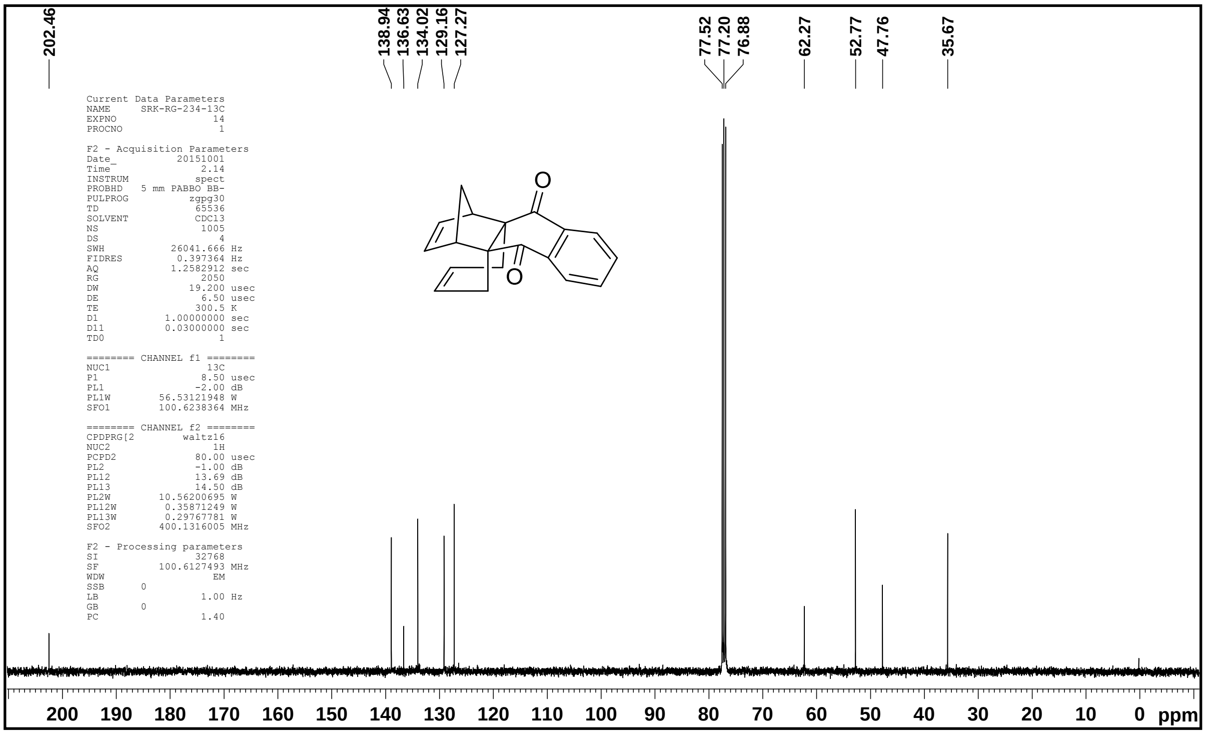

Compound **1aa'** (^{1}H NMR)

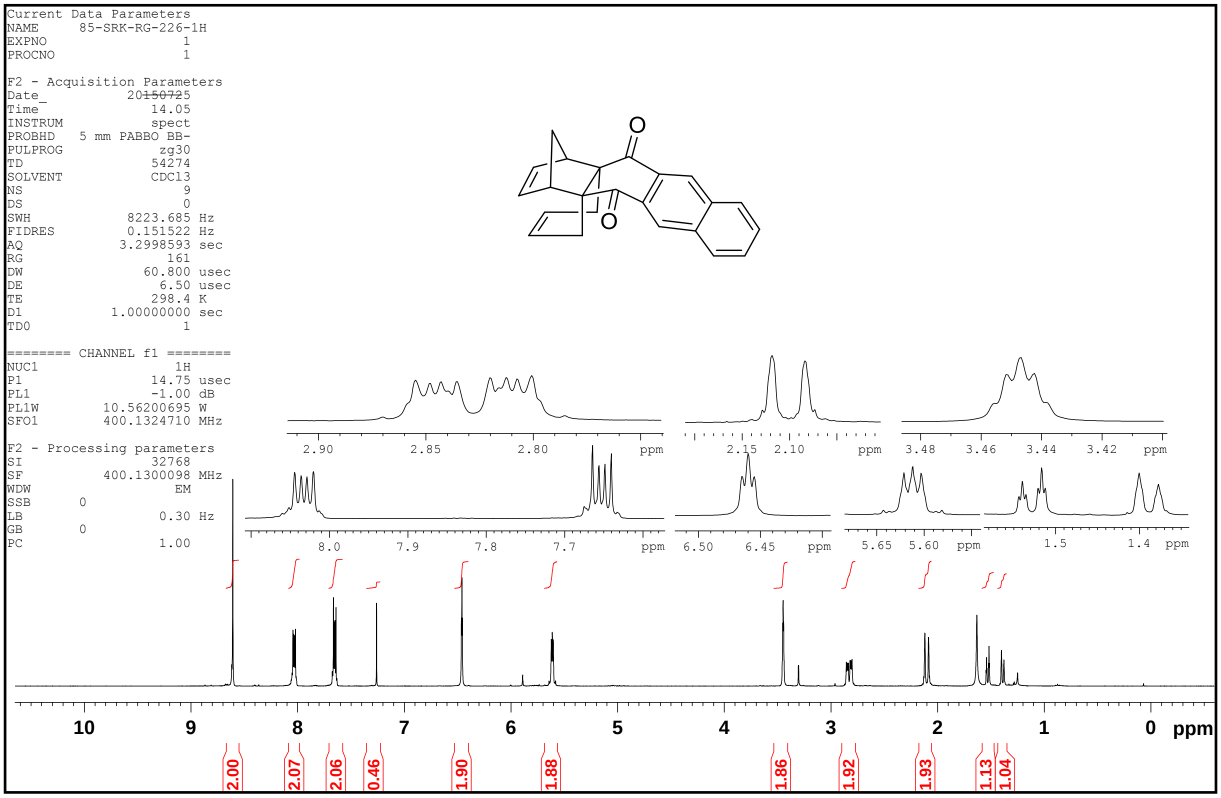

Compound **1aa'** (^{13}C NMR)

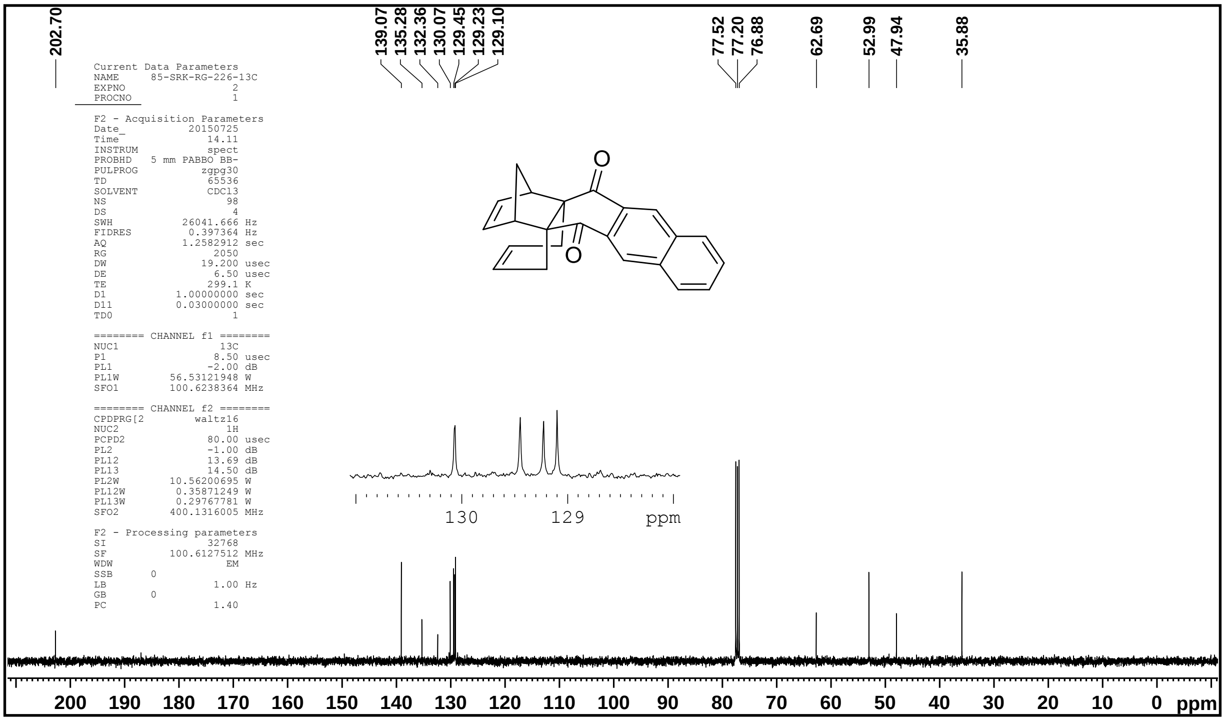

Compound **1aa'** (DEPT-135)

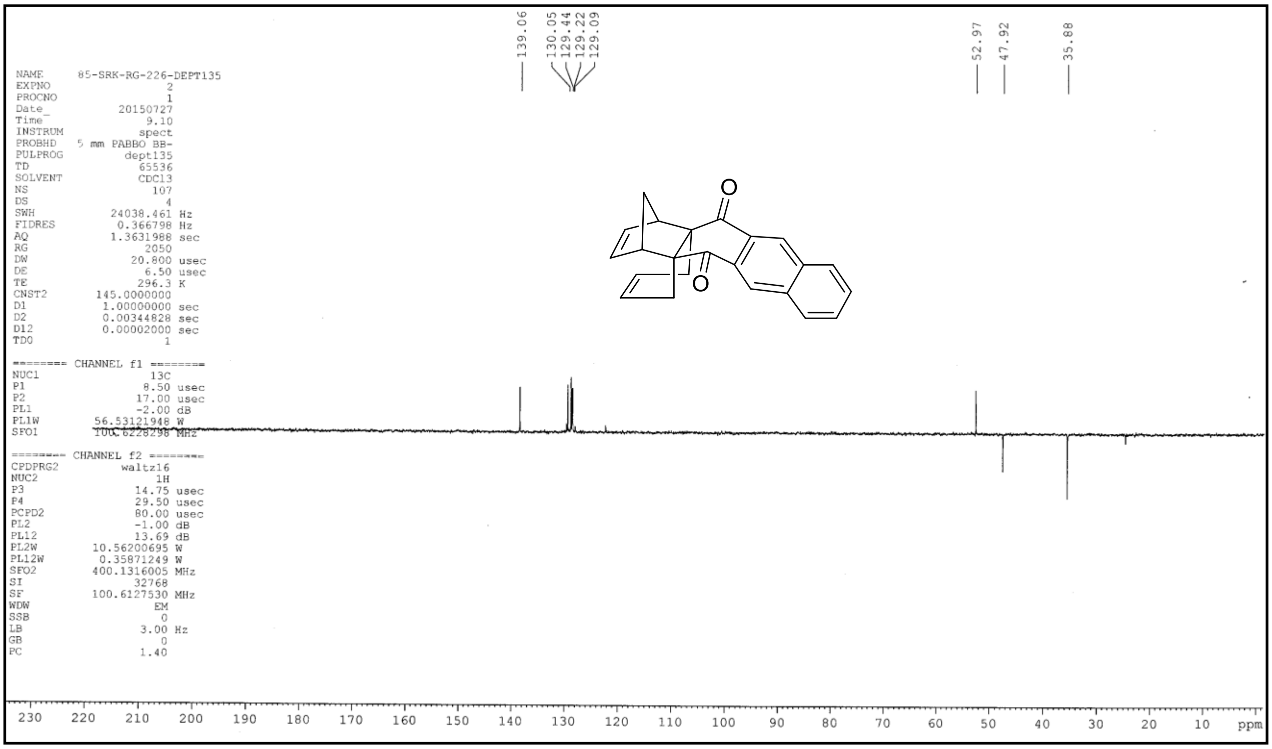

Compound **1b** (^{1}H NMR)

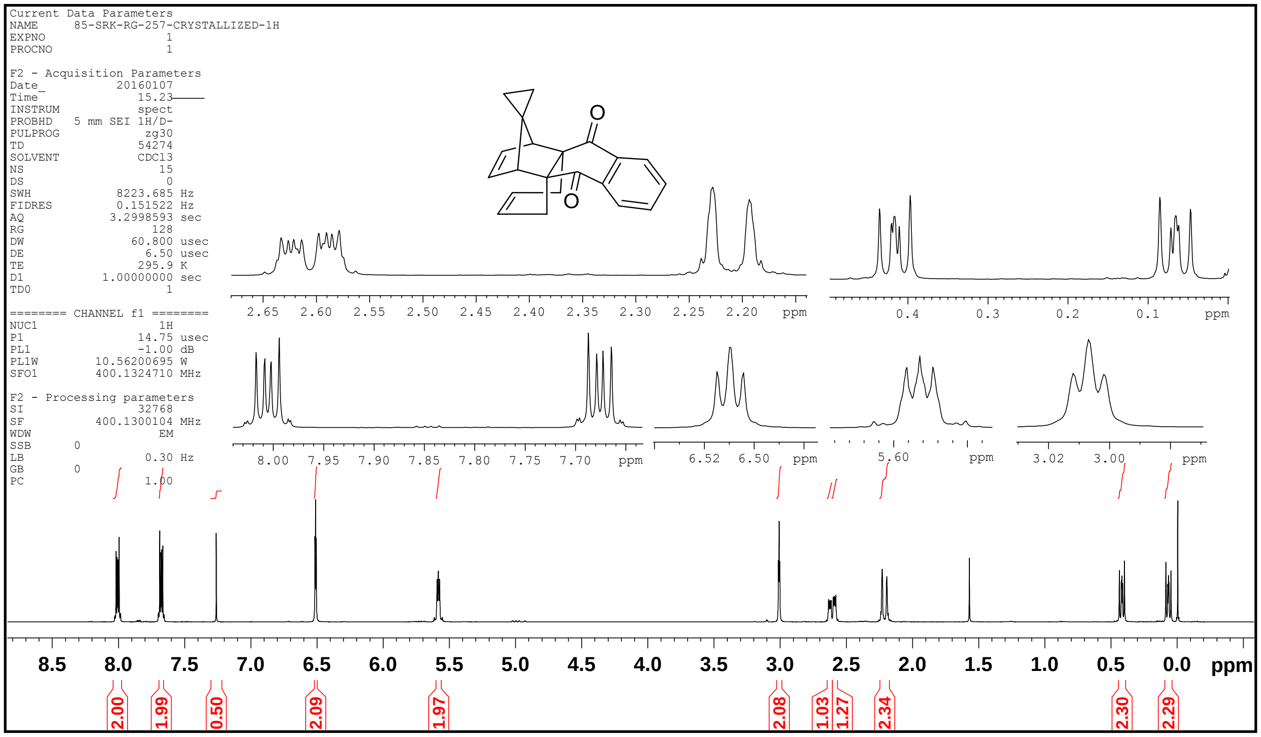

Compound **1b** (^{13}C NMR)

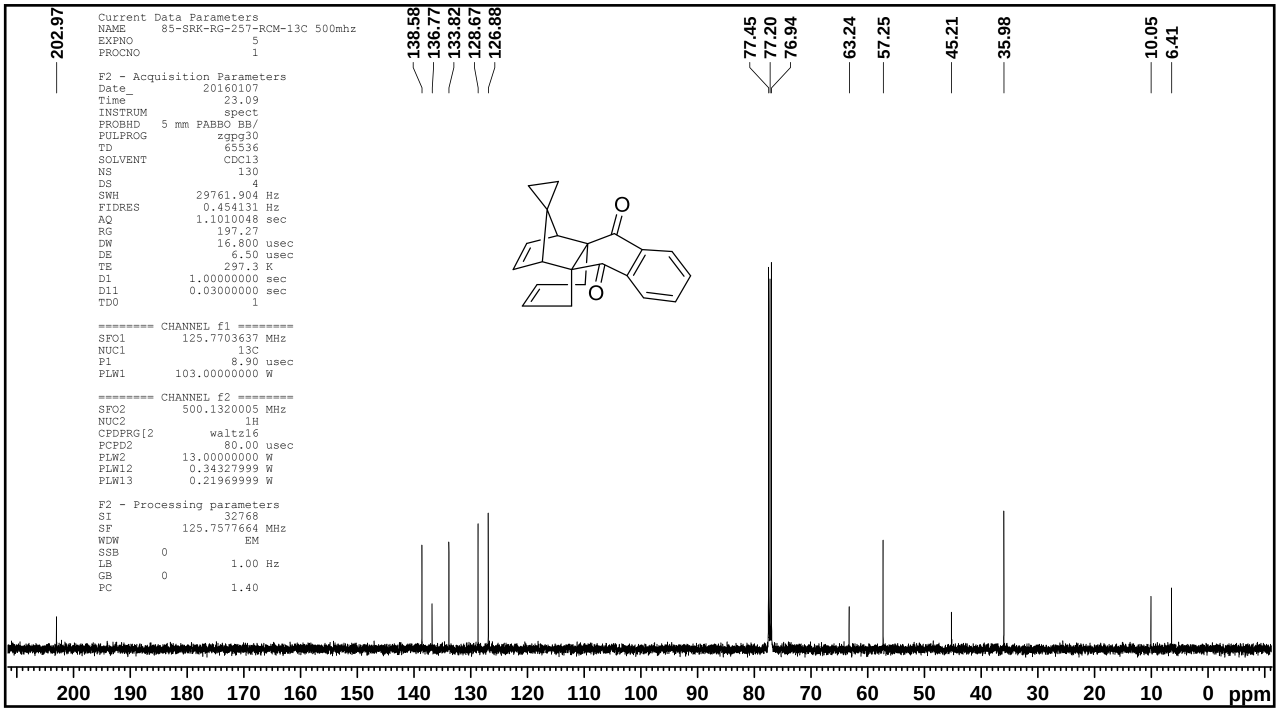

Compound **1b** (DEPT-135)

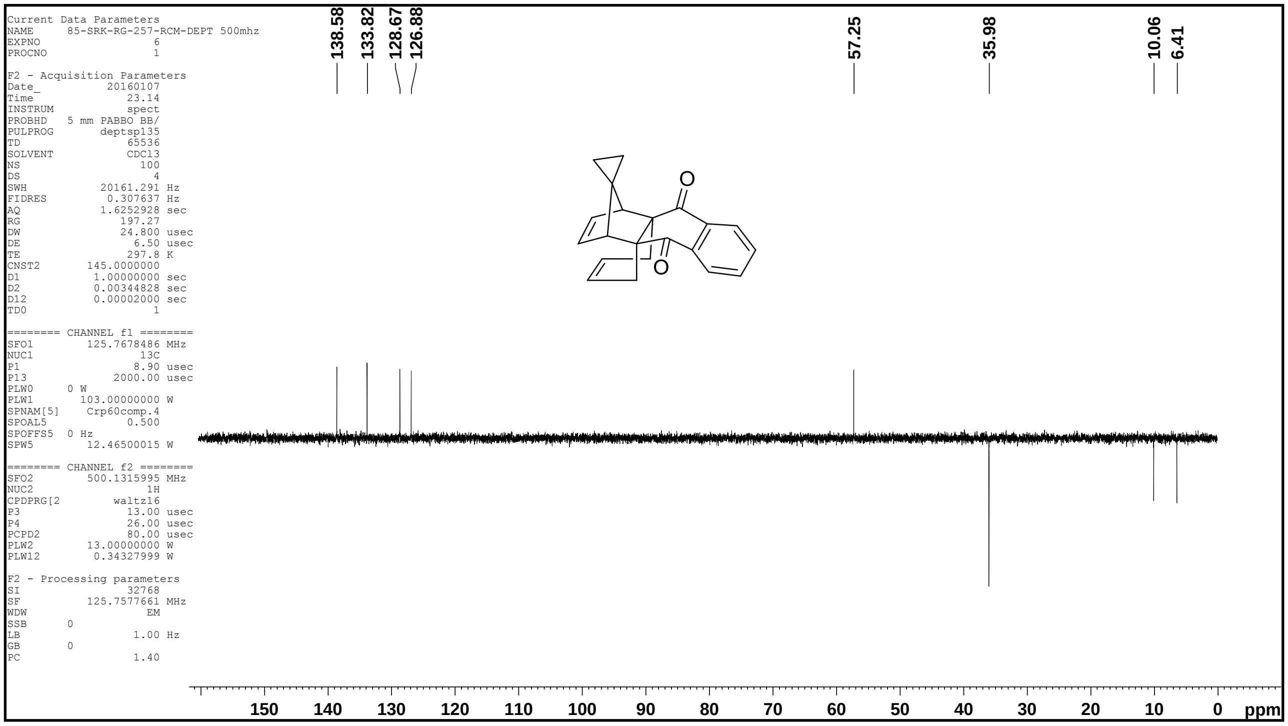

Compound **1bb'** (^{1}H NMR)

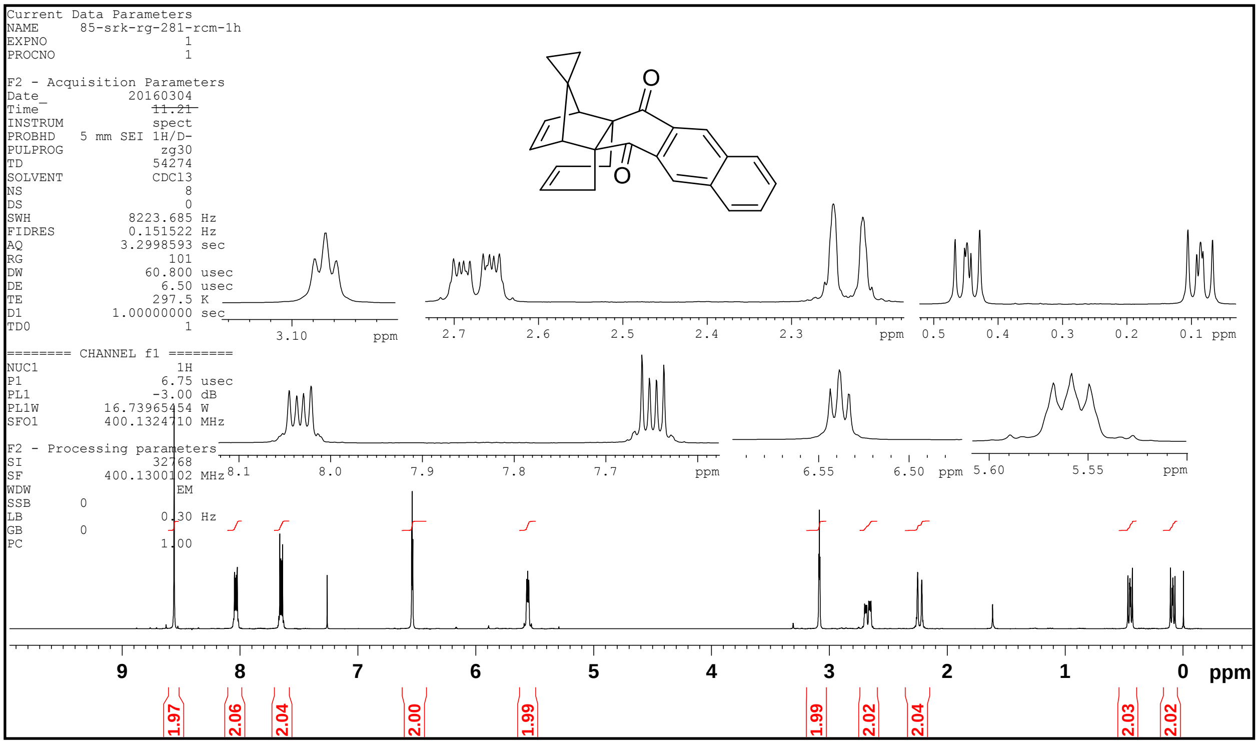

Compound **1bb'** (^{13}C NMR)

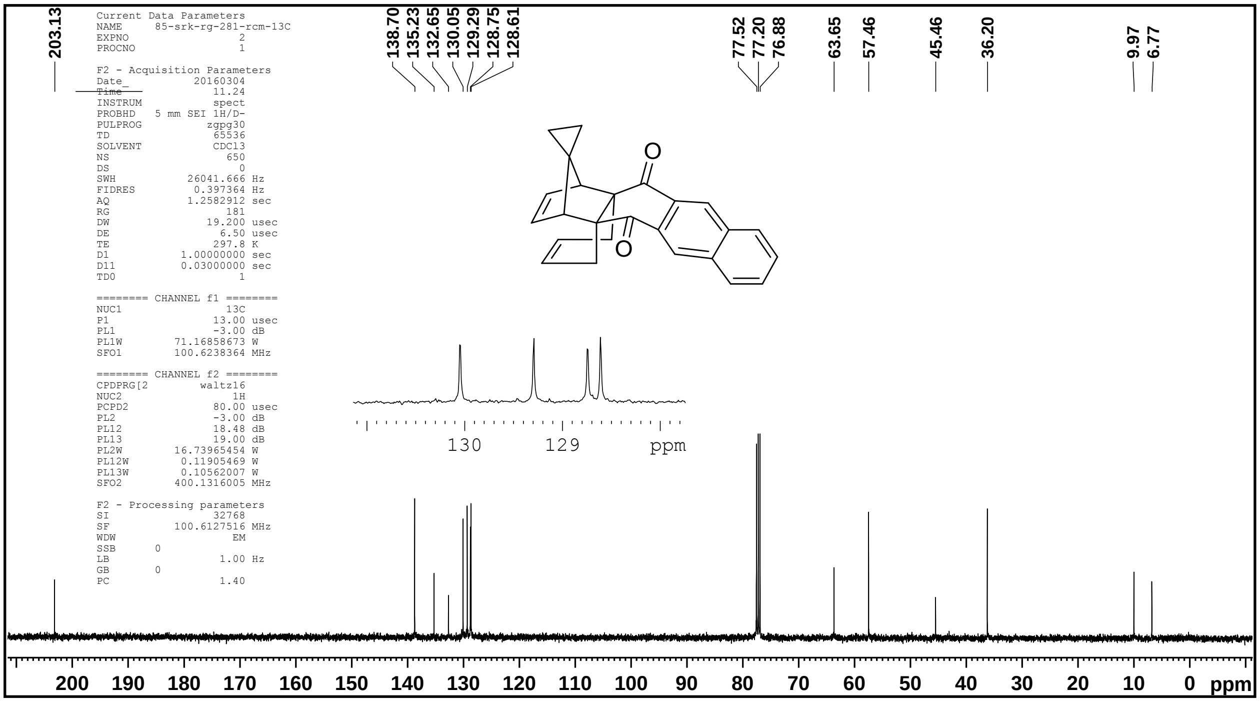

Compound **1bb'** (DEPT-135)

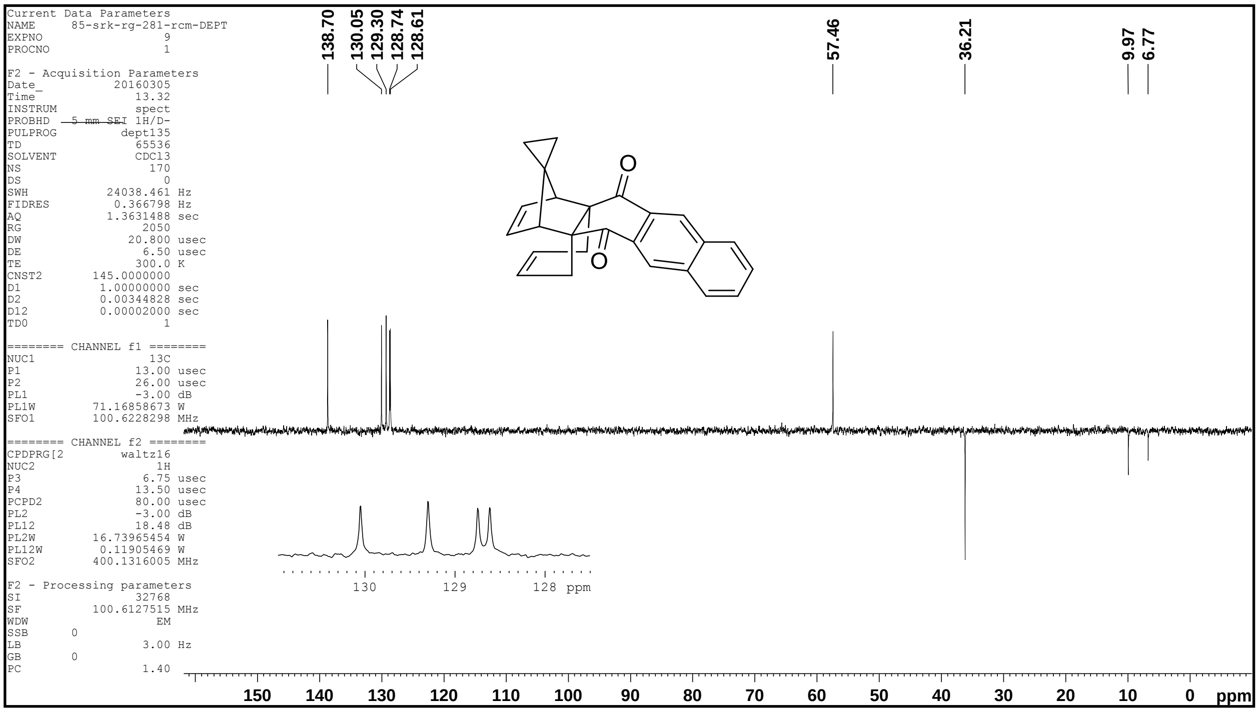

Compound **7** (^{1}H NMR)

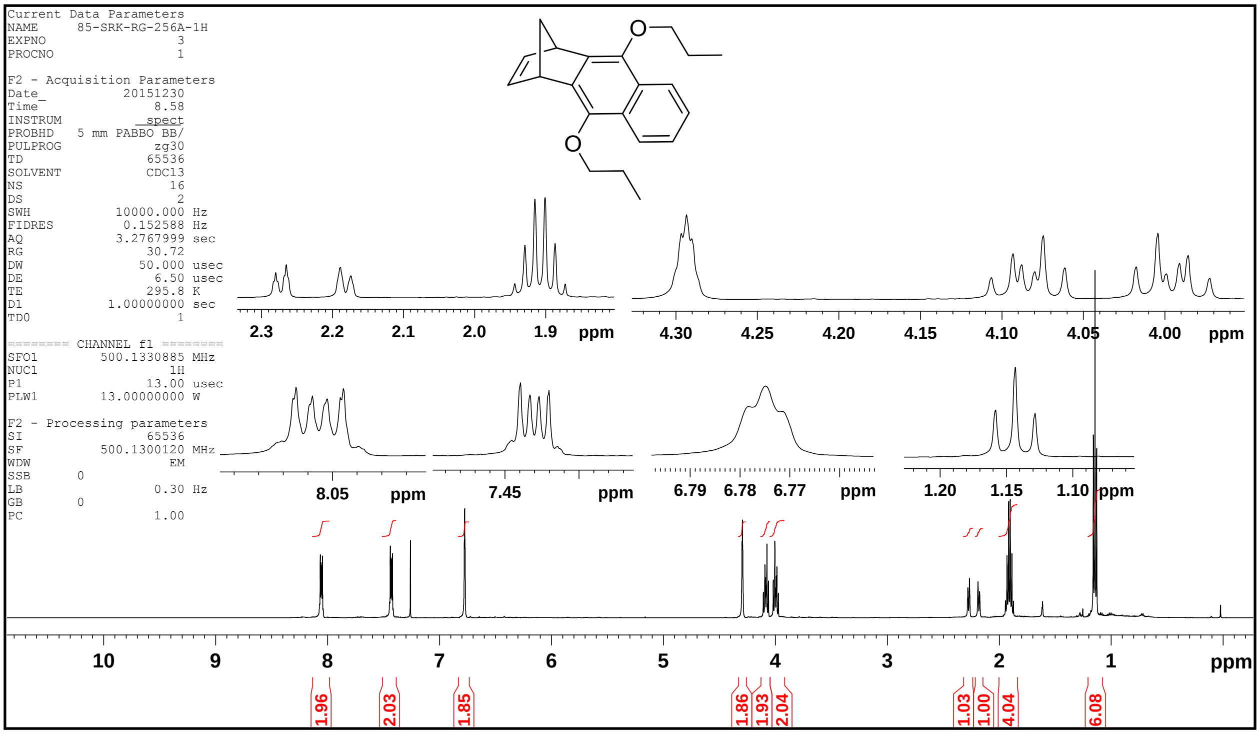

Compound **7** (^{13}C NMR)

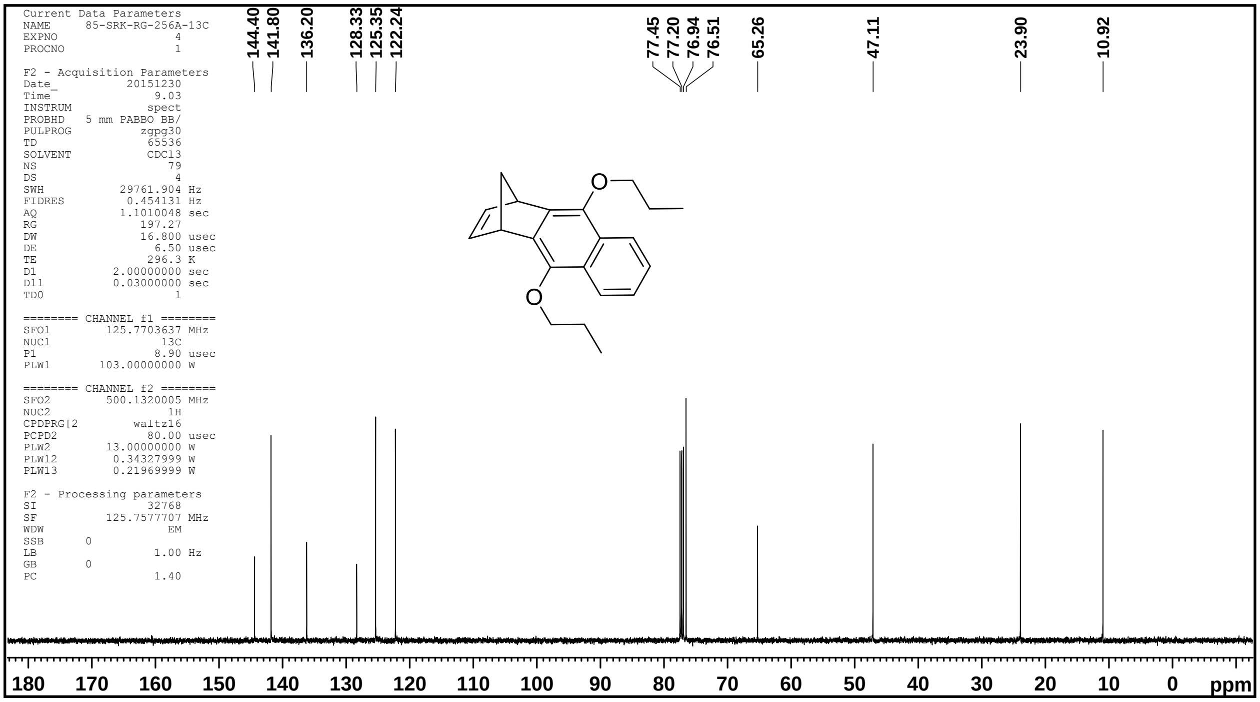

Compound **7** (DEPT-135)

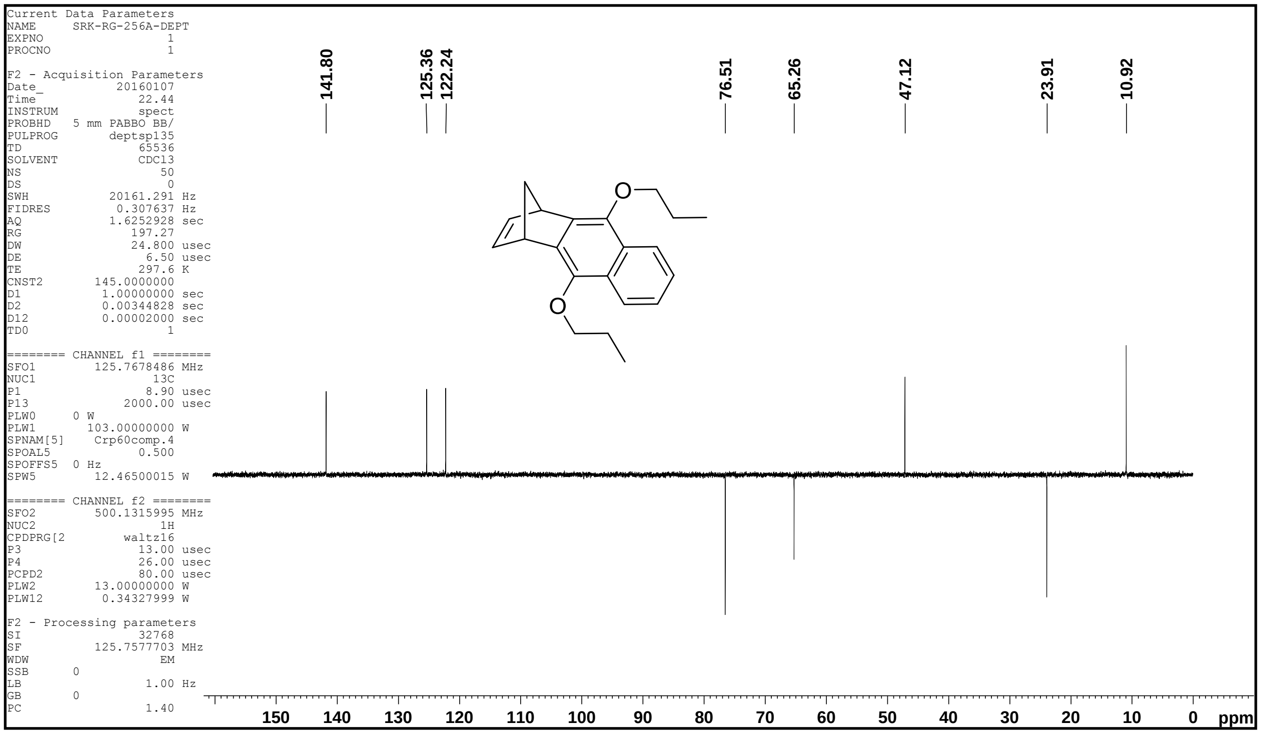

Compound **8** (^{1}H NMR)

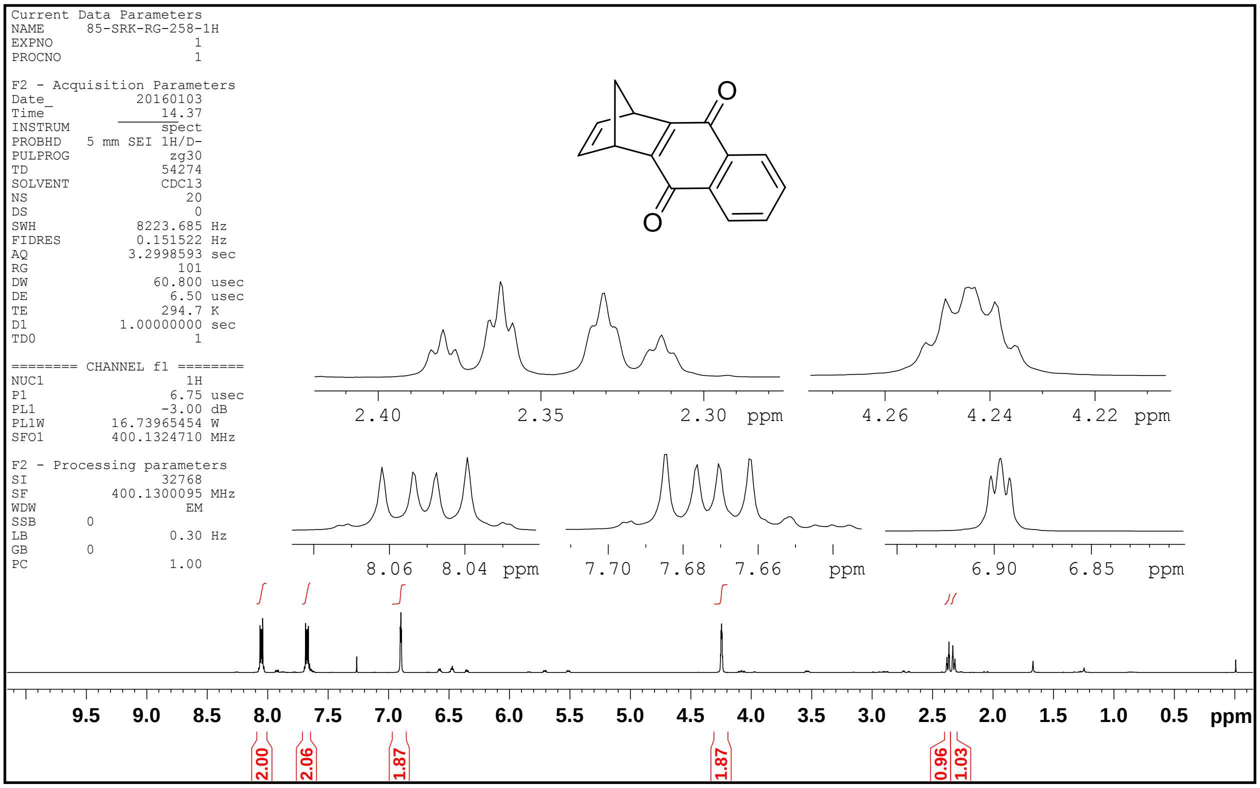

Compound **8** (^{13}C NMR)

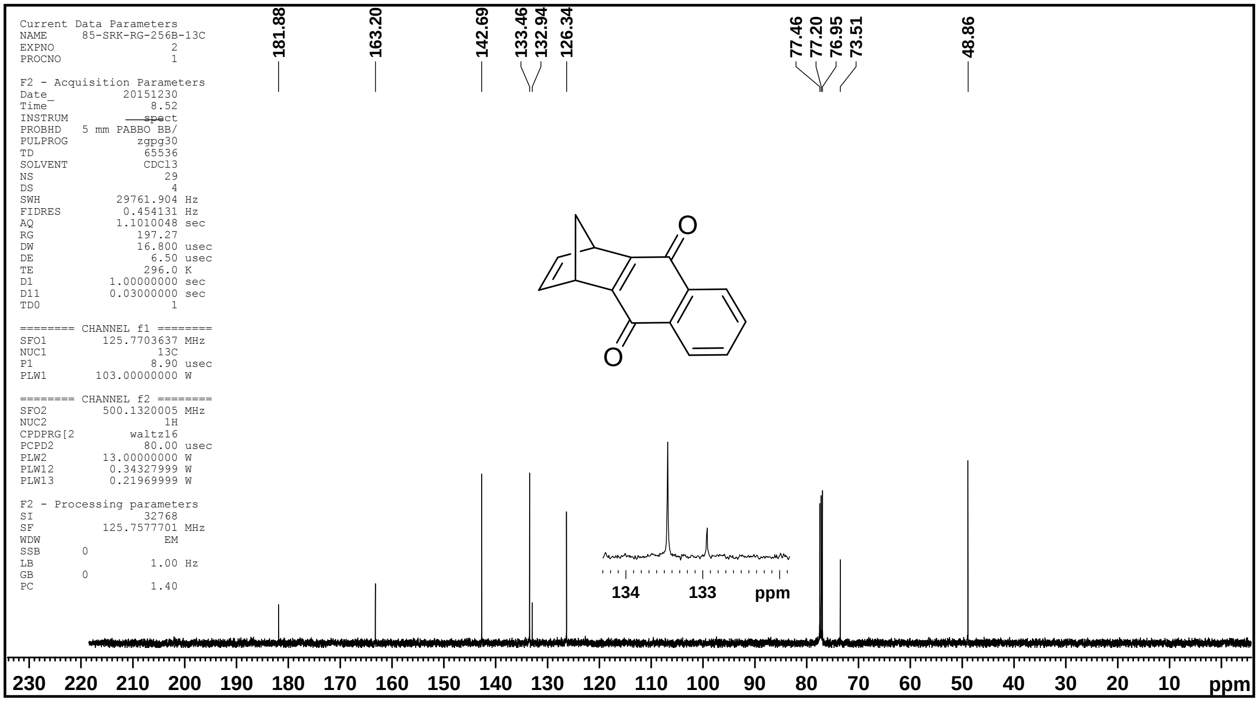

Compound **8** (DEPT-135)

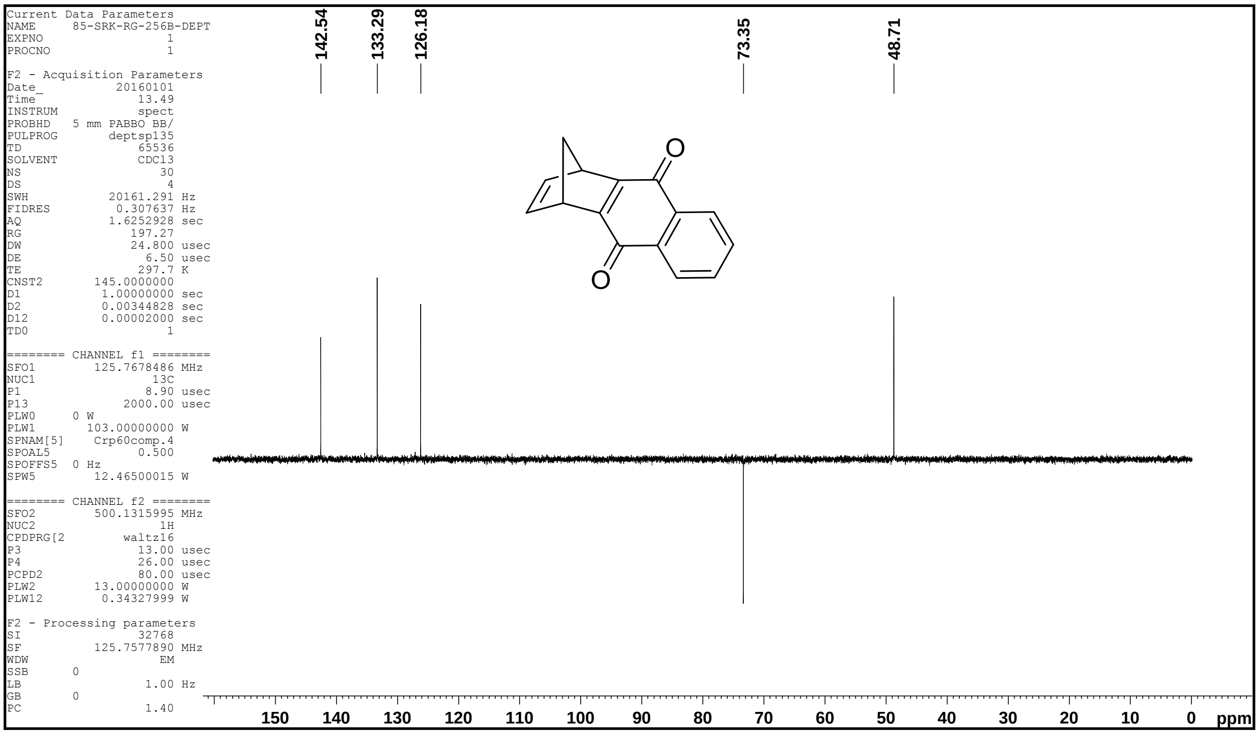

3. X-ray data for the compounds 1a, 1b, 2b and 15

X-ray data and refinement parameters for compound 1a (CCDC 1475412)

Bond precision:		C-C = 0.0040 Å	Wavelength = 0.71070
Cell:	a = 8.483(2)	b = 33.621(9)	c = 14.525(4)
	α = 90	β = 98.986(3)	γ = 90
Temperature: 100 K			
		**Calculated**	**Reported**
Volume		4091.8(18)	4091.8(18)
Space group		P 21/n	P 1 21/n 1
Hall group		-P 2yn	-P 2yn
Moiety formula		C_{19} H_{16} O_2	C_{19} H_{16} O_2
Sum formula		C_{19} H_{16} O_2	C_{19} H_{16} O_2
Mr		276.32	276.32
Dx,g cm^{-3}		1.346	1.346
Z		12	12
Mu (mm^{-1})		0.086	0.086
F000		1752.0	1752.0
F000′		1752.80	
h, k, lmax		10, 39, 17	10, 39, 17
Nref		7210	7185
Tmin, Tmax		0.965, 0.995	0.976, 0.993
Tmin′		0.934	

Correction method = # Reported T Limits: Tmin = 0.976 Tmax = 0.993

AbsCorr = NUMERICAL

Data completeness = 0.997 Theta(max) = 24.998

R(reflections) = 0.0650(5711) wR2(reflections) = 0.1544(7185)

S = 1.092 Npar= 568

X-ray data and refinement parameters for compound 1b (CCDC 1475453)

Bond precision:		C-C = 0.0028 Å	Wavelength=0.71070
Cell:	a = 7.524(3)	b = 8.498(3)	c =12.116(5)
	α = 88.457(13)	β = 79.090(1)	γ = 79.868(12)

Temperature: 150 K

	Calculated	Reported
Volume	748.8(5)	748.8(5)
Space group	P -1	P -1
Hall group	-P 1	-P 1
Moiety formula	$C_{21} H_{18} O_2$	$C_{21} H_{18} O_2$
Sum formula	$C_{21} H_{18} O_2$	$C_{21} H_{18} O_2$
Mr	302.35	302.35
Dx,g cm^{-3}	1.341	1.341
Z	2	2
Mu (mm^{-1})	0.085	0.085
F000	320.0	320.0
F000′	320.14	
h, k, lmax	8, 10, 14	8, 10, 14
Nref	2628	2618
Tmin, Tmax	0.986, 0.990	0.987, 0.993
Tmin′	0.982	

Correction method = # Reported T Limits: Tmin = 0.987 Tmax = 0.993

AbsCorr = NUMERICAL

Data completeness= 0.996	Theta(max) = 24.988
R(reflections)= 0.0429(1519)	wR2(reflections)= 0.1026(2618)
S = 0.840	Npar = 208

X-ray data and refinement parameters for compound 2b (CCDC 1475403)

Bond precision:	C-C = 0.0023 Å	Wavelength = 0.71075
Cell: a = 14.630(7)	b = 11.834(6)	c = 10.513(5)
α = 90	β = 90	γ = 90
Temperature: 150 K		

	**Calculated**	**Reported**
Volume	1820.1(15)	1820.1(15)
Space group	P n m a	P n m a
Hall group	-P 2ac 2n	-P 2ac 2n
Moiety formula	C_{23} H_{22} O_2	C_{23} H_{22} O_2
Sum formula	C_{23} H_{22} O_2	C_{23} H_{22} O_2
Mr	330.41	330.40
Dx, g cm^{-3}	1.206	1.206
Z	4	4
Mu (mm^{-1})	0.075	0.075
F000	704.0	704.0
F000′	704.31	
h, k, lmax	17, 14, 12	17, 14, 12
Nref	1693	1680
Tmin, Tmax	0.985, 0.985	0.784, 1.000
Tmin′	0.985	

Correction method = # Reported T Limits: Tmin = 0.784 Tmax = 1.000

AbsCorr = NUMERICAL

Data completeness = 0.992	Theta(max) = 24.991
R(reflections) = 0.0429 (1484)	wR2(reflections) = 0.1003 (1680)
S = 1.097	Npar = 118

X-ray data and refinement parameters for oxa-bowl/propellane hybrid (15) (CCDC 1451438)

Bond precision:	C-C = 0.0022 Å		Wavelength=0.71073
Cell:	a=8.1080(16)	b=24.504(5)	c=9.769(2)
	α = 90	β = 90	γ = 90
Temperature: 293 K			

	**Calculated**	**Reported**
Volume	1940.9(7)	1940.9(7)
Space group	P n a 21	P n a 21
Hall group	P 2c -2n	P 2c -2n
Moiety formula	$C_{27} H_{26} O_2$	$C_{27} H_{26} O_2$
Sum formula	$C_{27} H_{26} O_2$	$C_{27} H_{26} O_2$
Mr	382.48	382.48
Dx,g cm^{-3}	1.309	1.309
Z	4	4
Mu (mm^{-1})	0.081	0.081
F000	816.0	816.0
F000′	816.34	
h, k, lmax	11, 33, 13	11, 33, 13
Nref	5223[2758]	5070
Tmin, Tmax	0.971, 0.989	0.980, 0.991
Tmin′	0.971	

Correction method = # Reported T Limits: Tmin = 0.980 Tmax = 0.991

AbsCorr = NUMERICAL

Data completeness = 1.84/0.97 Theta(max) = 29.130

R(reflections) = 0.0414(4006) wR2(reflections) = 0.0931(5070)

S = 0.991 Npar = 262
